# Cohort Profile: The Stockholm Diabetes Prevention Programme (SDPP)

**DOI:** 10.1093/ije/dyac147

**Published:** 2022-07-12

**Authors:** Hrafnhildur Gudjonsdottir, Per Tynelius, Stefan Fors, Diego Yacamán Méndez, Mihretab Gebreslassie, Minhao Zhou, Axel C Carlsson, Pernilla Svefors, Per Wändell, Claes-Göran Östenson, Boel Brynedal, Anton Lager

**Affiliations:** Centre for Epidemiology and Community Medicine, Stockholm, Sweden; Department of Global Public Health, Karolinska Institutet, Stockholm, Sweden; Centre for Epidemiology and Community Medicine, Stockholm, Sweden; Department of Global Public Health, Karolinska Institutet, Stockholm, Sweden; Centre for Epidemiology and Community Medicine, Stockholm, Sweden; Aging Research Center, Karolinska Institutet & Stockholm University, Stockholm, Sweden; Centre for Epidemiology and Community Medicine, Stockholm, Sweden; Department of Global Public Health, Karolinska Institutet, Stockholm, Sweden; Centre for Epidemiology and Community Medicine, Stockholm, Sweden; Centre for Epidemiology and Community Medicine, Stockholm, Sweden; Division of Family Medicine and Primary Care, Department of Neurobiology, Care Sciences and Society, Karolinska Institutet, Huddinge, Sweden; Academic Primary Health Care Centre, Stockholm Region, Stockholm, Sweden; Department of Women’s and Children’s health, Uppsala University, Uppsala, Sweden; Division of Family Medicine and Primary Care, Department of Neurobiology, Care Sciences and Society, Karolinska Institutet, Huddinge, Sweden; Department of Molecular Medicine and Surgery, Karolinska Institutet, Stockholm, Sweden; Centre for Epidemiology and Community Medicine, Stockholm, Sweden; Department of Global Public Health, Karolinska Institutet, Stockholm, Sweden; Centre for Epidemiology and Community Medicine, Stockholm, Sweden; Department of Global Public Health, Karolinska Institutet, Stockholm, Sweden

Key Features

- The Stockholm Diabetes Prevention Programme (SDPP) was established in the mid-1990s as a baseline for a community-based intervention aimed at primary prevention of type 2 diabetes (T2D). The intervention was found to be ineffective, but the cohort continues to contribute to our understanding of the pathogenesis of T2D and cardiometabolic risk factors.
- The cohort comprises 15 070 men and 19 416 women, born between 1938 and 1961, resident in five municipalities in Stockholm County, Sweden, at baseline. A sub-cohort answered a screening survey (10 236 men and 16 481 women), and a sub-cohort of those participated in a clinical examination (3128 men and 4821 women) at baseline (clinical cohort).
- The clinical cohort has been followed up after 10 years, when 2383 men and 3329 women participated, and after 20 years, when 1752 men and 2545 women participated.
- Socioeconomic, demographic and health-related register information was collected for all. The screening survey contains self-reported information on own and familial T2D. For the clinical cohort, we conducted oral glucose tolerance tests, drew blood and took blood pressures and anthropometric measurements. The participants also filled in questionnaires on lifestyle and psychosocial conditions.
- Data are available on request after ethical approval; information is available on the study webpage [https://www.ces.regionstockholm.se/var-verksamhet/aktiviteter-och-projekt/sdpp/].

## Why was the cohort set up?

The Stockholm Diabetes Prevention Programme (SDPP) was established in the mid-1990s as a baseline for a community-based intervention aimed at the primary prevention of prediabetes and type 2 diabetes (T2D). The secondary research aims included mapping the links between family history of type 2 diabetes (FHD) and the risk of developing T2D; the role of genetic and other factors in the development of hyperglycaemia; and the development of cardiometabolic risk factors throughout the life course. The health care authorities of Region Stockholm initiated the study together with three departments at the medical university Karolinska Institutet. Region Stockholm, which continues to fund and manage the cohort, is the administrative and political body responsible for nearly all funding of health care in the county and most of its operation.

The intervention’s aim was to affect modifiable behavioural risk factors for T2D at the population level, focusing on the age range within which T2D most often manifests. Therefore, all inhabitants aged 35–56 years from five municipalities in Stockholm—three intervention and two control areas—were identified from population registers and included in the cohort. The intervention, consisting of a range of local bottom-up health promotion initiatives (e.g. invitations to walking groups, brochures with recipes for healthy food, and helping tobacco users to quit smoking), aimed to reduce the incidence of T2D by 25% over a 10-year period.^1^ After a first follow-up, the interventions were deemed unsuccessful. However, the longitudinal data collection continued to gather data on T2D, hyperglycaemia and cardiometabolic conditions and their risk factors. Over time, the SDPP has become an essential resource for research and health policy development. This cohort profile focuses on the data collection procedure and subsequent research and not on the initial, unsuccessful, intervention.

## Who is in the cohort?

The SDPP is a population-based, prospective cohort study comprising all men born between 1938 and 1957 (*n *= 15 070) and all women born between 1942 and 1961 (*n *= 19 416) who lived in selected municipalities at the time of the baseline study, which was 1992–94 for men and 1996–98 for women, making the participants 35–56 years old at baseline. This age range was selected to maximize the proportion of individuals at risk of developing T2D during the initially planned study period. The first four municipalities were Sigtuna, Tyresö, Upplands-Bro and Värmdö, all located on the outskirts of Stockholm. Sigtuna and Värmdö were included as intervention municipalities, as they were already participating in a healthy diet intervention programme funded by Region Stockholm, and Tyresö and Upplands-Bro were included as control municipalities. An additional intervention municipality, Upplands Väsby, was included for women at the request of one of Stockholm’s Health and Medical Service Districts involved in the planning of the study. The five selected municipalities were similar in size, with 21 000–35 000 inhabitants, and had similar demographic structures.^1^^,^^2^

The 34 486 identified men and women in the targeted age range were sent a one-page screening survey by mail. The full SDPP cohort (*n* = 34 486) includes two sub-cohorts. The first sub-cohort consists of those who responded to the one-page screening survey. The survey was completed by 79% of the men (*n *= 10 236 out of 12 952) and 85% of the women (*n* = 16 481 out of 19 416). This sub-cohort is referred to as the ‘survey responders’ (*n *= 26 717). Nearly 2% of them reported already having diabetes (*n *= 510).

**Figure 1:**
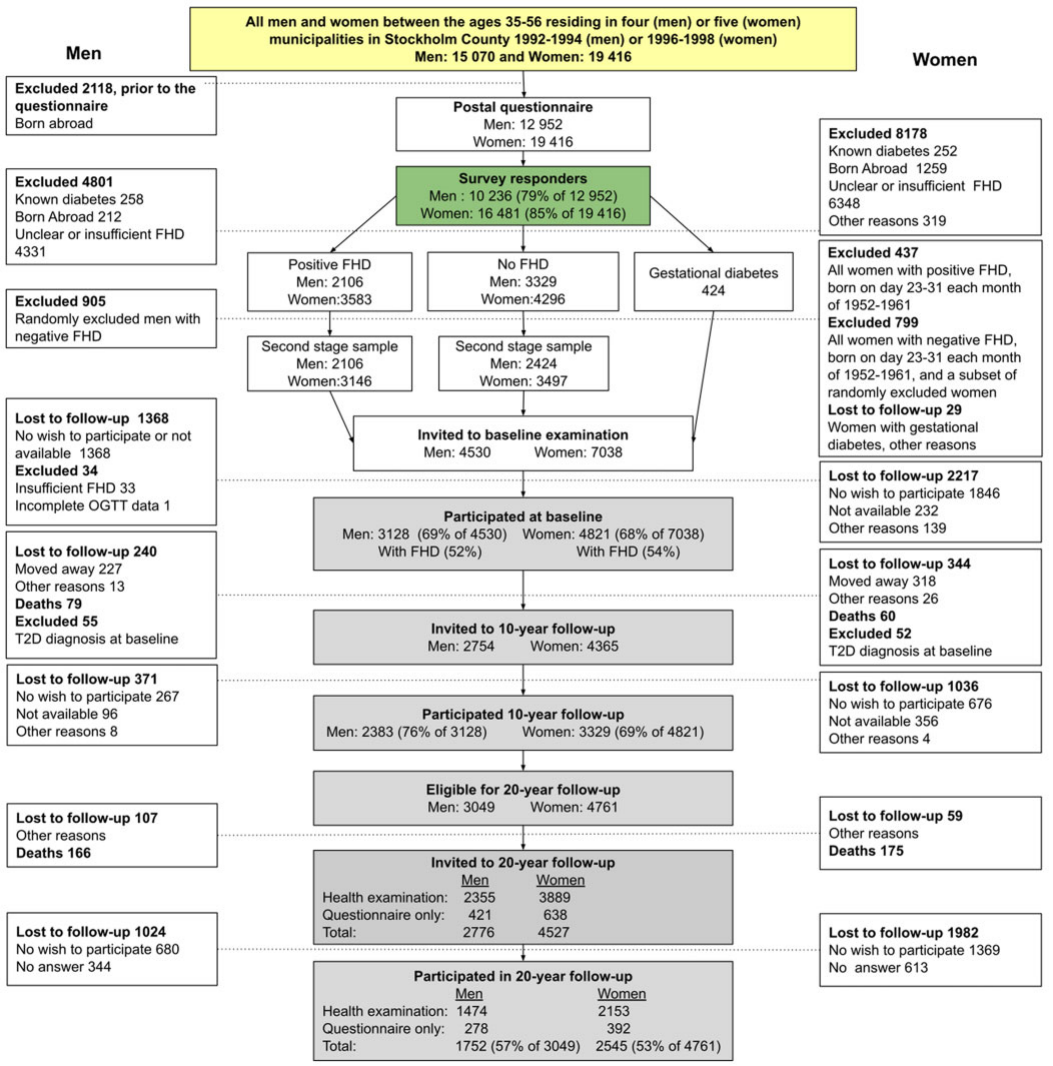
Flowchart of the Stockholm Diabetes Prevention Programme (SDPP) cohorts and selection process. Different parts of the cohort are indicated by colour, where yellow = full cohort, green = survey responders, grey = clinical cohort. T2D, type 2 diabetes; FHD, family history of type 2 diabetes; OGTT, oral glucose tolerance test

From the survey responders, we selected individuals without a current T2D diagnosis to follow longitudinally. Further, since the effect exerted by FHD was of interest, we selected individuals who had either strong, or complete absence, of FHD (see Figure 1). Strong FHD was defined as having at least one first-degree relative (parents, brother, sister or child) or two second-degree relatives (grandparents, aunts or uncles) with T2D. Absence of FHD was defined as having none of the above nor any first cousins with T2D. Those with intermediate or unknown FHD were excluded. Additionally, to limit bias in genetic studies we selected individuals who were born in Sweden. Further, all women with a history of gestational diabetes were invited. Next, all women born on Day 23–31 of each month of 1952–61, without a history of gestational diabetes, were excluded due to budget restrictions. A randomly selected sample of men and women without FHD were also excluded by age and municipality to balance the number of participants with and without FHD.

**Figure 2:**
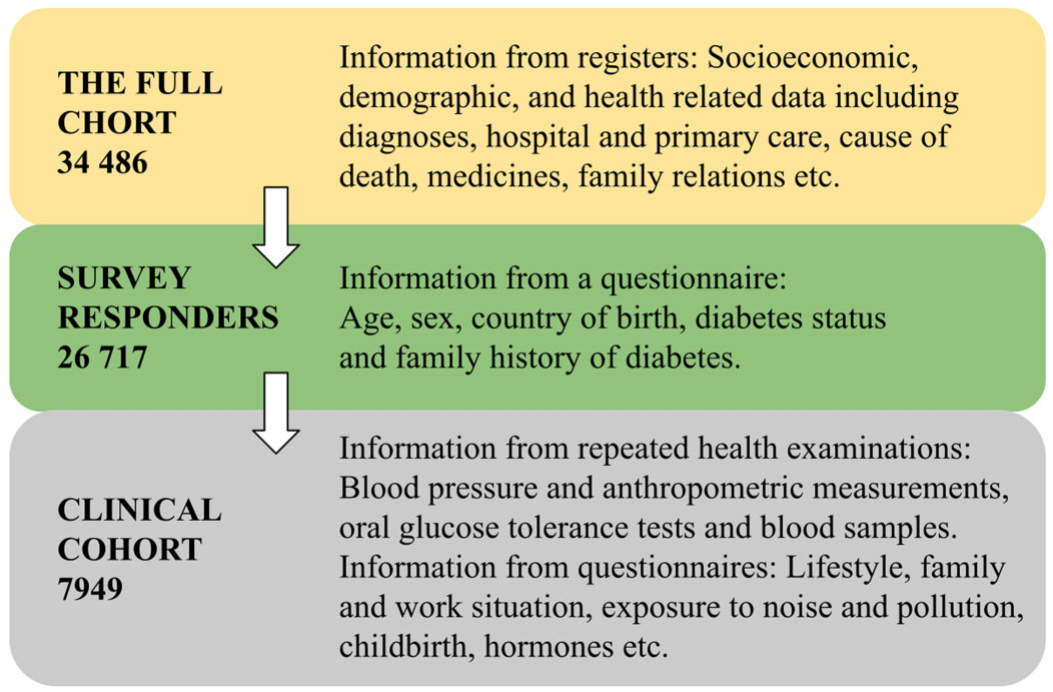
Overview of the number of participants and information in the Stockholm Diabetes Prevention Programme (SDPP) cohorts

Based on these selection criteria, 11 568 individuals were invited to a clinical examination. Almost 69% of the men (*n* = 3128 out of 4530) and 68% of the women (*n* = 4821 out of 7038) participated in the clinical examinations. These individuals formed what is referred to as the ‘clinical cohort’ (*n *= 7949). Compared with the full cohort, owing to the selection process just described, the clinical cohort is enriched with a higher proportion of individuals with and without FHD and only includes people born in Sweden. The sociodemographic characteristics of the full cohort and the clinical cohort at baseline, the population in Stockholm County and Sweden, are presented in Table 1. Figure 2 provides an overview of the number of participants and information available on the full cohort and sub-cohorts.

**Table 1:** Sociodemographic characteristics of men born 1938–57 and women born 1942–61 in the clinical cohort at baseline, the full cohort, and the population in Stockholm County and Sweden in 1992^a^

	Clinical cohort	Full cohort	Stockholm County	Sweden
	*n* = 7949 (%)	*n* = 34 486 (%)	*n* ^b^ = 479 000 (%)	*n* ^b^ = 2 293 000 (%)
Highest attained education				
University (>12 y)	32.5	28.4	35.4	27.0
Upper secondary school 3 y^c^ (12 y)	15.3	15.0	15.3	12.4
Upper secondary school 2 y^d^ (11 y)	33.2	34.2	28.7	33.2
Primary school (9 y)	19.0	22.4	20.6	27.3
	*P*1 <0.001; *P*2 <0.001	*P*1 <0.001; *P*2 <0.001	*P*1 <0.001	
Occupation				
Higher non-manual employees	26.4	22.4	22.2	19.9
Intermediate non-manual employees	22.4	20.8	17.9	15.3
Lower non-manual employees	11.3	12.5	11.6	15.7
Skilled workers	15.6	18.3	16.4	24.2
Unskilled workers	8.3	11.4	13.2	12.0
Other	16.0	14.7	18.7	13.0
	*P*1 <0.001; *P*2 <0.001	*P*1 <0.001; *P*2 <0.001	*P*1 <0.001	
Income, quintiles^e^				
5 (highest)	28.8	26.1	29.4	20.0
4	22.4	21.3	20.5	20.0
3	19.2	19.0	18.5	20.0
2	16.8	17.5	15.8	20.0
1 (lowest)	12.9	16.1	15.9	20.0
	*P*1 <0.001; *P*2 <0.001	*P*1 <0.001; *P*2 <0.001	*P*1 <0.001	
Birth country				
Sweden	100	85.0	79.9	88.2
	*P*1 <0.001; *P*2 <0.001	*P*1 <0.001; *P*2 <0.001	*P*1 <0.001	

y, years.

a Data from integrated database for labour market research 1992.

b Age- and sex-matched samples according to the Stockholm Diabetes Prevention Programme (SDPP) municipalities distributions.

c 3 years of upper secondary school are required for university studies in Sweden.

d 2 years of upper secondary school gave vocational qualifications in Sweden until the 90 s.

e Income quintiles according to age- and sex-matched samples from the Swedish total population in 1992.

*P1 = P-v*alue compared with Sweden; *P2 = P-va*lue compared with Stockholm County.

## How often have they been followed up?

The clinical cohort has been followed up with new clinical examinations twice after the baseline examination. The first follow-up was conducted 10 years after the baseline examination for the men (2383 participated) and 8 years after for the women (3329 participated). This data collection wave is referred to as the 10-year follow-up. The second follow-up was conducted on average 22 years after the baseline examination for the men (1752 participated) and 19 years after that for the women (2545 participated). This data collection wave is referred to as the 20-year follow-up.

Migration from Stockholm County and non-response were the main causes of attrition in the first and second clinical follow-ups (see Figure 1). To increase the participation rate, two reminders were sent by mail, and those who failed to respond to these reminders were contacted by phone. In the lead-up to the 20-year follow-up, a questionnaire including questions about diabetes diagnoses since the baseline study was included with the second reminder, to minimize complete non-response for those not participating in this clinical examination.

The baseline characteristics of participants in the clinical cohort who participated in the 20-year follow-up, and of those lost to follow-up, are presented in Table 2. Those lost to follow-up were more likely to:have prediabetes or T2D; smoke be obese; and exhibit higher blood pressure than those who participated in the 20-year follow-up. They were also more likely to: have lower education levels; be manual workers; and come from less advantaged economic backgrounds than those who participated.

**Table 2:** Baseline characteristics of participants in the clinical cohort that participated in the 20-year follow-up and those lost to follow-up

	Men		Women	
	Followed	Lost to follow-up	Followed	Lost to follow-up
	% (*n*)	% (*n*)	% (*n*)	% (*n*)
	*n* = 1752	*n* = 1376	*n* = 2545	*n* = 2275
Age				
≥45 years	67.9 (1190)	68.7 (945)	75.6 (1924)	70.2 (1596)
		*P* <0.001		*P* <0.001
Highest attained education				
University (>12 y)	25.5 (445)	20.8 (285)	41.7 (1060)	31.8 (723)
Upper secondary school 3 y^a^ (12 y)	46.6 (811)	40.6 (556)	32.8 (833)	33.8 (768)
Upper secondary school 2 y^b^ (11 y)	6.1 (107)	5.6 (77)	8.7 (220)	7.2 (163)
Primary school (9 y)	21.8 (379)	33.0 (452)	16.9 (430)	27.2 (618)
		*P* <0.001		*P* <0.001
Occupational status				
Higher non-manual employees	24.6 (421)	16.2 (216)	17.6 (435)	9.9 (220)
Intermediate non-manual employees	26.2 (449)	22.2 (297)	32.3 (797)	26.4 (588)
Lower non-manual employees	16.6 (284)	15.5 (207)	25.9 (639)	26.2 (582)
Skilled workers	15.8 (271)	21.3 (285)	9.7 (239)	13.0 (290)
Unskilled workers	11.3 (193)	18.0 (240)	11.0 (272)	20.0 (446)
Other	5.5 (94)	6.9 (92)	3.6 (88)	4.4 (99)
		*P* <0.001		*P* <0.001
Economic situation				
Able to procure 1500 €^c^	94.8 (1647)	89.6 (1217)	84.3 (2145)	76.0 (1728)
Smoking				
Never	42.0 (736)	34.4 (473)	38.7 (985)	33.2 (754)
Former	36.6 (641)	33.6 (462)	40.5 (1029)	33.4 (760)
Current	21.4 (374)	32.0 (440)	20.8 (529)	33.4 (759)
		*P* <0.001		*P* <0.001
Physical activity				
Sedentary	9.4 (164)	12.2 (168)	10.3 (261)	12.3 (279)
Moderate	50.2 (879)	54.8 (753)	53.0 (1349)	60.5 (1375)
Regular	31.4 (549)	24.7 (340)	29.2 (742)	20.6 (467)
Active	9.1 (159)	8.2 (113)	7.5 (191)	6.6 (151)
		*P* <0.001		*P* <0.001
BMI (kg/m^2^)				
<18.5	0.1 (2)	0.5 (7)	0.8 (21)	1.2 (28)
18.5–24.9	43.4 (758)	36.8 (506)	56.2 (1424)	48.8 (1106)
25.0–29.9	46.8 (818)	47.6 (654)	32.9 (833)	33.6 (763)
≥30.0	9.6 (168)	15.1 (207)	10.1 (257)	16.4 (371)
		*P* <0.001		*P* <0.001
FHD				
Yes (positive)	50.0 (876)	54.1 (745)	53.9 (1371)	53.2 (1210)
		*P* = 0.001		*P* = 0.002
OGTT				
Type 2 diabetes	1.4 (25)	2.9 (40)	0.9 (22)	1.8 (41)
Prediabetes	5.4 (95)	9.7 (133)	3.4 (87)	5.3 (121)
Normal	93.2 (1632)	87.4 (1203)	95.7 (2436)	92.9 (2113)
		*P* <0.001		*P* <0.001
OGTT and FHD				
Type 2 diabetes if positive FHD	2.1 (18)	4.4 (33)	1.4 (19)	2.1 (26)
Prediabetes if positive FHD	6.1 (53)	12.8 (95)	4.7 (64)	6.3 (76)
Normal if positive FHD	91.9 (805)	82.8 (617)	93.9 (1288)	91.6 (1108)
		*P* <0.001		*P* = 0.060
Type 2 diabetes if negative FHD	0.8 (7)	1.1 (7)	0.3 (3)	1.4 (15)
Prediabetes if negative FHD	4.8 (42)	6.0 (38)	2.0 (23)	4.2 (45)
Normal if negative FHD	94.4 (827)	92.9 (586)	97.8 (1148)	94.4 (1005)
		*P* = 0.496		*P* <0.001

BMI, body mass index; y, years; OGTT, oral glucose tolerance test; FHD, family history of type 2 diabetes.

a 3 years of upper secondary school are required for university studies in Sweden.

b 2 years of upper secondary school gave vocational qualifications in Sweden until the 90 s.

c Able to procure ≈1500 euros in 1 week, according to 2014 monetary value.

For the full cohort (34 486 individuals), extensive follow-up data are available from the regional and national registers. Since the information on deaths is shared nationally as well as between countries, complete loss to follow-up (censoring) was limited to the few individuals who had migrated abroad and were still alive.

## What has been measured?

The data were gathered from: health examinations, questionnaires and blood samples during visits to health care centres, for the clinical cohort; questionnaires distributed by mail during the recruitment period, for the survey responders; and extensive data from regional and national registers, for the full cohort.

Table 3 provides an overview of the data collected from the clinical health examinations for each follow-up phase in the clinical cohort. During visits to the health care centre, blood pressure was measured along with anthropometric measurements, and a standard 75-g oral glucose tolerance test (OGTT) was administered. Blood samples were taken, and whole blood (0-h samples) and blood serum (0-h and 2-h samples) were stored at −20°C (baseline) and −80°C (10-year and 20-year follow-up) for future analyses of insulin and genetic and cardiometabolic biomarkers. The whole blood and the blood serum samples are currently stored at −80°C. Participants additionally completed an extensive questionnaire (Table 3) that included questions concerning lifestyle, family and work situation and other psychosocial conditions. The women answered additional questions regarding childbirth, contraceptive medications, menstrual status and hormonereplacement therapy and gestational diabetes. During the follow-up studies, the participants answered additional questions regarding noise, pollution, personality traits, stress, financial situation, sleeping habits and social networks.

**Table 3:** Collected data for each follow-up phase in the clinical cohort

Variables	Baseline	10-year follow-up	20-year follow-up
**Health examination data**			
Height	x	x	x
Weight	x	x	x
Waist and hip circumference	x	x	x
Blood pressure	x	x	x
Blood samples	x	x	x
Oral glucose tolerance test (OGTT)	x	x	x
**Questionnaire data**			
Born in Sweden	x	x	x
Diabetes			
Diabetes diagnoses	x	x	x
Family history of diabetes	x	x	x
Gestational diabetes	x	x	x
Background			
Marital status	x	x	x
Household composition	x	x	x
Children	x	x	x
Financial capacity	x	x	x
Education	x	x	x
Parental occupation	x		
Siblings	x	x	
Childhood circumstances	x	x	
Lifestyle			
Diet	x	x	x
Physical activity	x	x	x
Smoking habits	x	x	x
Tobacco use (oral moist tobacco, snus)	x	x	x
Alcohol	x	x	x
Body weight and management			
Birthweight	x		
Intentional weight loss	x	x	x
Weight history	x	x	x
Parental weight history	x	x	x
General health	x	x	x
Cognitive health			x
Inventory of Interpersonal Problems (IIP)		x	x
Health-related Personality Inventory		x	x
Psychological distress	x	x	x
Sense of coherence	x	x	x
Social contacts	x	x	x
Psychosocial work environment			
Work pressure	x	x	x
Work tempo	x	x	x
Demand-control	x	x	x
Work shifts	x	x	x
Type of work	x	x	x
Sleeping habits			x
Retirement			x
Hormone therapy			
Contraceptives	x	x	
Hormone replacement therapy	x	x	x
Reproductive history			
Menstruation	x	x	x
Pregnancy	x	x	x
Erectile function			x
Noise		x	x

Those within the clinical cohort who had reported diabetes diagnoses since the previous clinical follow-up did not undergo additional OGTT. Instead, fasting blood samples were taken, and information on the year of diagnosis and treatment was collected.

For the survey responders, data collected via the screening survey are available, including age, sex, country of birth, diabetes status, FHD and gestational diabetes.

Information from the registers for the full cohort includes selected variables on socioeconomic, demographic, geographical and health-related data on morbidity and mortality (Table 4). The Swedish national and regional registers are regularly updated by linkage, using the unique personal identity number assigned to all residents allowed to stay in Sweden for at least 1 year.^3^ The National Board of Health and Welfare administers the national health registers, whereas Statistics Sweden administers registers containing demographic information and census data. Most of these registers (including registers on family relations, educational attainment, occupation, employment status, income, diagnoses from hospitalizations, pregnancies and births, and causes of death) had full coverage already from the time of or prior to the SDPP cohort’s establishment. Other national registers have since achieved full, or near full, coverage.^3^

**Table 4:** Overview of register linkages in the SDPP cohort

Data provider	Register/database	Available information
Register Centre Västra Götaland, Sweden	National Diabetes Register	Has an 87% coverage. Includes information on year of diagnosis, physical activity, blood pressure, HbA1c, BMI, smoking habits, lipids, diabetes duration, complications, and treatment^4^
National Board of Health and Welfare	National Patient Register	Covers almost 100% of inpatient care from 1987 and onwards and about 80% of the specialized outpatient doctor visits from private and public caregivers since 2001^3^
	Swedish Medical Birth Register	Includes data from 1973 from prenatal care (e.g. pregnancies, age and smoking habits), delivery care (e.g. fetal presentation, mode of delivery, analgesia and anaesthesia) and neonatal care (e.g. anthropometric measures, duration of pregnancy, and Apgar scores)^3^
	Swedish Cancer Register	Data from 1958 onwards; the overall completeness and data quality are very high. Contains data on selected confirmed tumours, site of tumour, histological type, stage and date of diagnosis^3^
	Swedish Cause of Death Register	Data from 1961 onwards; includes the underlying and contributory causes of death and date of death among individuals registered in Sweden at the time of death^3^
	National Prescribed Drug Register	Includes data from 2005 onwards on selected dispensed prescription drugs in Sweden (e.g. date of prescribing and dispensing, amount, brand name, formulation and the package of the dispensed item)^3^
Statistics Sweden	Longitudinal integration database for health insurance and labour market studies (LISA)	Includes sociodemographic data from 1990 onwards on all individuals aged 16 years and older (e.g. country of birth, education, occupation, employment status, income and family income, sick leave and disability pension)^27^
	National Census in Sweden 1960, 1965, 1970, 1975, 1980, 1985, 1990, 2011	Includes individual and household data (e.g. educational level, occupational status, income, housing and size and type of household)^28^
	Geodatabase	Includes information from 1982 onward on regional divisions and coordinates of properties in Sweden^28^
	Multi-generation Register	Includes data from 1961 onwards and on those born in 1932 or later. Links the index persons to their biological or adoptive parents, allowing us to identify first- and second-degree relatives^28^
	Total Population Register	Includes data from 1968 onwards, e.g. on births, immigration and emigration, deaths, civil status and citizenship.^3^
Region Stockholm	Stockholm Regional Healthcare Data Warehouse (VAL)	Includes data from 1997 onwards on health care utilization from primary and specialist outpatient care and in-hospital care. Includes diagnoses and service provider information^5^
	Stockholm County Council Public Health surveys from 2002, 2006, 2007, 2010, and 2014	Includes extensive data on randomly selected individuals aged 18 and older living in Region Stockholm. (e.g. health-related data and lifestyle, perinatal, familial, socioeconomic, demographic factors)^29^
Military archives	Military conscription register	Includes data on men who underwent military conscription at age 18 from 1969 onwards (e.g. BMI, blood pressure, fitness and cognitive tests)^30^

BMI, body mass index.

One of SDPP’s further strengths is the linkage to the Swedish National Diabetes Register, which includes data on all patients with a diabetes diagnosis, irrespective of who provided the health care, from large university hospitals to primary health care centres.^4^ This register is particularly useful, given that T2D is often handled at the primary health care level in Sweden and cases may be missing from the older hospital discharge register.

For all participants living in Stockholm County, the cohort is also linked to the Stockholm Regional Healthcare Data Warehouse (VAL), regional databases with data on diagnoses and health care use.^5^ In these databases, the coverage of inpatient diagnoses is complete for the whole study period. Diagnoses from specialist outpatient care are partially available from 2001 and completely from 2013, and diagnoses from primary care are partially available from 2003 and completely from 2013. The linkage of register data to the SDPP cohorts facilitates further studies of health complications and consequences due to T2D and cardiometabolic factors over time.

## What has it found?

SDPP has hitherto contributed to 107 published articles and, entirely or partly, to 24 doctoral theses. A list of these publications and doctoral theses is available on the study webpage [https://www.ces.regionstockholm.se/var-verksamhet/aktiviteter-och-projekt/sdpp/]. Most of the publications thus far are based on analysis of data collected at baseline and at the 10-year follow-up among the clinical cohort. These analyses have contributed to our understanding of factors that impact on T2D risk. These factors include FHD,^6^ weight history,^7^ low birthweight,^8^ tobacco use [smoking and the use of oral moist tobacco (snus)],^9^ alcohol consumption,^10^ coffee consumption,^11^ psychological distress,^12^ work stress and sense of coherence,^13^ personality,^14^ socioeconomic position,^15^ aircraft noise^25^ and factors predicting normalization of glucose intolerance.^16^ Several studies have explored genes associated with T2D and biomarkers for T2D and obesity.^17^^,^^18^ Furthermore, the data have contributed to international studies on the relationship between air pollution and noise, respectively, and later risk of hypertension, obesity, cancer, cardiovascular diseases and mortality.^19–22^

Additional data from the 20-year follow-up of the clinical cohort and register linkages for the full cohort provide opportunities for a wide range of future research avenues. Recently, a study on life course trajectories of weight and T2D risk was published.^23^ Several studies are ongoing, including one with cluster analysis for risk stratification of T2D, one on fruit and vegetable intake and T2D risk and one on socioeconomic inequalities in blood pressure, cardiometabolic factors and undiagnosed T2D.

Based on two clinical follow-ups and register linkage to the National Diabetes Register and the Stockholm Regional Healthcare Data Warehouse (VAL), we have identified 1339 individuals with T2D diagnoses since the baseline examination (Table 5). The cumulative incidence of T2D within the clinical cohort has therefore been 17.1% since baseline, when 7820 men and women were without T2D diagnosis.

**Table 5:** Number of type 2 diabetes (T2D) diagnoses within the clinical cohort since the examination at baseline, years 1992–96

	*n*
Without T2D at baseline (disease free)	7820
T2D diagnoses	
Data source	
Stockholm Diabetes Prevention Programme 10-year follow-up	288
Stockholm Diabetes Prevention Programme 20-year follow-up^a^	394
National Diabetes Register^b^	624
Stockholm Regional Healthcare and Utilization Data (VAL)^c^	33
Total number of T2D diagnoses since the baseline examination	1339
Cumulative incidence	17.1%

a Until February 2018.

b Until June 2021.

c Until June 2020.

**Figure 3:**
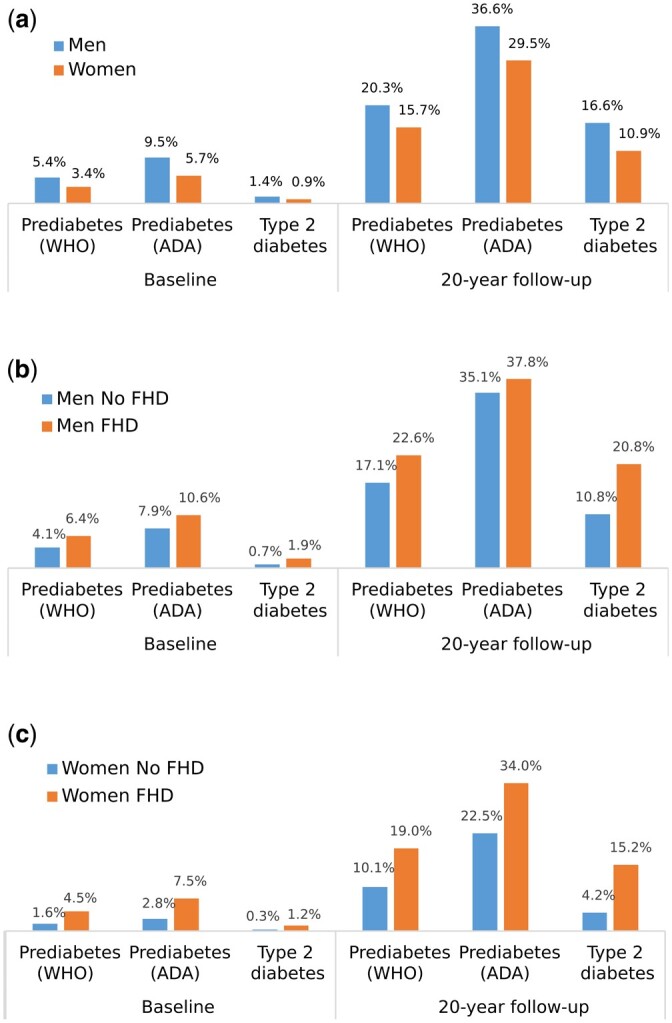
Prediabetes and type 2 diabetes prevalence at baseline and at 20-year follow-up within the clinical cohort among: a) men and women; b) men with and without family history of type 2 diabetes (FHD); and c) women with and without FHD. Prediabetes is defined according to the American Diabetes Association (ADA) 2004 and World Health Organization (WHO) 1999

Figure 3a illustrates the prevalence of prediabetes and T2D among the men and women in the clinical cohort at baseline and at the 20-year follow-up. Figure 3b and c illustrates the prevalence among men and women with and without FHD. Men had a higher prevalence of prediabetes and T2D than women, especially at the 20-year follow-up (Figure 3a). A higher T2D prevalence was observed among men and women with FHD than those without FHD (Figure 3b and c). This association was mainly observed at the 20-year follow-up, when twice as many individuals with FHD were diagnosed with T2D. The prevalence of prediabetes was also higher among women with FHD than among women without FHD, especially at the 20-year follow-up.

## What are the main strengths and weaknesses?

The SDPP’s clinical cohort provides extensive clinical, biological and self-reported data from up to three clinical examinations over a period of 20 years. In addition to high participation rates in these follow-ups, the data contain detailed sociodemographic and health-related information from registers for the full cohort, including individuals that dropped out from or never attended the clinical examinations. Thus, the register data allow total data investigation of the reasons for not attending, reasons that very well may be associated with T2D or cardiovascular risk, such as alcohol abuse. We believe that few other datasets provide similar possibilities. Additionally, information from the registers regarding parental education, occupation and income facilitates investigations of the associations between early life conditions and later health outcomes, in the clinical cohort. Furthermore, with regional data from inpatient and outpatient care, including primary care, we have truly comprehensive data on health care usage and relevant diagnoses, for the entire cohort during their residency in Stockholm County. Detailed information is also available for everyone who received a diabetes diagnosis, irrespective of region, thanks to the linkage to the Swedish National Diabetes Register. Since countries share information on deaths, the definitive loss to follow-up is low across the entire cohort, in principle limited to those who emigrated from Sweden and have not yet died.

Based on the same sex-specific age ranges as the clinical cohort at baseline, Stockholm County had higher proportions of highly educated people, high-income earners and non-manual workers, in comparison with the general Swedish population (Table 1). The clinical cohort showed similar distributions to those of Stockholm County, although the occupational gradient was somewhat steeper.

A study from 2011 compared data from the SDPP baseline screening with data from the National Prescribed Drug Register, and concluded that the risk of T2D was similar among participants and non-participants.^24^ However, as T2D is more common among individuals of lower socioeconomic status,^25^ the T2D incidence in the clinical cohort could be lower than in the general Swedish population. Furthermore, the exclusion of foreign-born individuals and the oversampling of participants with and without FHD in the clinical cohort may limit generalizations from the clinical cohort to the national population.

## Can I get hold of the data? Where can I find out more?

The SDPP study group welcomes collaborations on studies that will enhance our understanding of T2D and cardiometabolic risk factors. Researchers and research groups are encouraged to use SDPP data to address new research questions. Data are available on request after ethical approval. Information and instructions for applicants are available on the study webpage [https://www.ces.regionstockholm.se/var-verksamhet/aktiviteter-och-projekt/sdpp/], along with a list of publications. For further information, please contact the study principal investigator, Anton Lager, at [anton.lager@regionstockholm.se].

## Ethics approval

The SDPP cohort study was conducted in accordance with the ethical principles outlined in the Declaration of Helsinki.^26^ The routines concerning consent and ethical vetting have developed over time in Sweden. In the first wave for men and women, and the second wave for men, informed oral consent was obtained and register linkage was not yet planned. For women in the second wave and for all participants in the third wave, informed written consent was obtained. In the third wave, the information to participants, as well as the subsequent consent, included information about register linkages. All this was carefully described for the Swedish Ethics Review Authority before their approval 2019–06531. That approval complemented two prior approvals from the regional ethics review board of Stockholm (2013/1982–31/2, 2018/2345–32).

## Author contributions

H. G. wrote the manuscript’s first draft. All authors provided critical revisions and contributed relevant intellectual content. All authors approved the final version of the manuscript.

## Funding

The cohort is supported and funded by Region Stockholm.
